# Protein-Engineered Large Area Adipose-derived Stem Cell Sheets for Wound Healing

**DOI:** 10.1038/s41598-018-34119-x

**Published:** 2018-10-26

**Authors:** Jongbeom Na, Seung Yong Song, Jae Dong Kim, Minsu Han, June Seok Heo, Chae Eun Yang, Hyun Ok Kim, Dae Hyun Lew, Eunkyoung Kim

**Affiliations:** 1 Department of Chemical and Biomolecular Engineering, Yonsei University, 50 Yonsei-ro, Seodaemun-gu, Seoul 03722 South Korea; 2 Institute for Human Tissue Restoration, Department of Plastic & Reconstructive Surgery, Yonsei University College of Medicine, Seoul, South Korea; 3 Cell Therapy Center, Severance Hospital, Yonsei University College of Medicine, Department of Laboratory Medicine, Yonsei University, 50 Yonsei-ro, Seodaemun-gu, Seoul 03722 South Korea

## Abstract

Human adipose-derived stem cells (hADSCs) formed robust cell sheets by engineering the cells with soluble cell adhesive molecules (CAMs), which enabled unique approaches to harvest large area hADSC sheets. As a soluble CAM, fibronectin (FN) (100 pg/ml) enhanced the cell proliferation rate and control both cell-to-cell and cell-to-substrate interactions. Through this engineering of FN, a transferrable hADSC sheet was obtained as a free-stranding sheet (122.6 mm^2^) by a photothermal method. During the harvesting of hADSC sheets by the photothermal method, a collagen layer in-between cells and conductive polymer film (CP) was dissociated, to protect cells from direct exposure to a near infrared (NIR) source. The hADSC sheets were applied to chronic wound of genetically diabetic *db*/*db* mice *in vivo*, to accelerate 30% faster wound closure with a high closure effect (ε_wc_) than that of control groups. These results indicated that the engineering of CAM and collagens allow hADSC sheet harvesting, which could be extended to engineer various stem cell sheets for efficient therapies.

## Introduction

Chronic wounds, including chronic non-healing wounds, have become a serious problem^1^ due to diabetes, arterial or venous insufficiency, and radiation-induced damage. These chronic wounds can induce secondary damage, such as infections, impairment and aberrant extracellular matrix accumulation^2,3^. The conditions of chronic wounds are associated with multifactorial processes that significantly contribute to dysfunctional wound healing^4,5^. To resolve these problems, many approaches have been attempted, such as debridement, antibiotic therapy, tissue-engineered materials, and cell-based therapy^2,5^. Among them, cell-based therapy has been revealed as a new method for the treatment of chronic wounds^6,7^. Particularly, human adipose-derived stem cells (hADSCs), a type of mesenchymal stem cell (MSC), have shown various advantages for wound healing, such as a fast rate of healing, self-renewal capacity, wound contraction reduction, and paracrine activity^8,9^. Furthermore, hADSCs secrete favorable angiogenic factors, thus highlighting their capability to induce skin neovascularization^10,11^.

However, the conventional transplantation of stem cells involves syringe-based injection following the process of detachment from the culture plate and re-suspension, which is prone to exhibiting low engraftment efficiency due to rapid diffusion, limited localization to the host tissue, damage from enzymatic digestion, and shear-induced cell death. In addition, other challenges facing stem cell therapy in the functional recovery of damaged tissue include maintaining the unlimited proliferation of stem cells and selectively activating lineage-dependent cell signaling^12,13^. Therefore, a universal approach to effective cell therapy is desirable for the engraftment of cells.

Cell sheet technology^14^ has been a topic of interest for its potential applicability in cell engineering^15,16^ including wound healing^17,18^. In particular, previous studies have revealed that the transplantation of ADSC sheets combined with artificial skin showed significant wound-healing effects after 2 weeks of treatment^19^. Traditionally, cell sheets were prepared from temperature-responsive culture dishes by covalently grafting poly(*N*-isopropylacrylamide (NIPAAm)) onto a dish surface; the cell sheet was detached from the surface at a lower temperature (20 ~ 4 °C)^20^. However, these pNIPAAm-based cooling methods for the preparation of cell sheets possess two conflicting technical challenges: (1) cells with low affinity need long culture times to reach a confluent monolayer, and (2) cells with high affinity require a longer time for detachment (τ_d_) at low temperatures (reportedly requiring τ_d_ > 60 min)^21–23^. With these limitations, it seems quite difficult to retain the original cell-cell interactions.

To overcome these limitations, we recently developed a fast photothermal method of fibroblast cell sheet preparation that was capable of producing various cell sheet patterns with multiple sheet production capacity within τ_d_ of <5 min^24–26^. The advantages of photothermal (PT) method over pNIPAAm-based (NP) method using thermo-responsive polymers could be summarized as follows: (1) the sheet harvesting time (few minutes) for PT method is much faster than the NP method (~30 min)^27^, (2) the harvesting condition for PT method is at room temperature while the NP method is at cold temperature (~20 °C)^28^, (3) the sheet harvesting could be controlled spatially in PT method and more precisely controlled as optical resolution is higher than thermal resolution. As this method offers a non-invasive means of cell sheet harvesting, it could be applied to stem cell sheets, such as hADSC sheets for wound healing.

However, the proliferation of stem cells and cell sheet formation require widespread adhesion to the substrate^29^, and this adhesion is extremely sensitive to the microenvironment^30,31^. For example, cellular adhesion and the proliferation of cells are known to vary by the collagen thickness of the substrate^31^. Furthermore, spreading morphology is necessary for the proliferation of stem cells to prevent anoikis arising from the loss of cell-substrate interactions. Thus, the formation of free-standing or transferrable ADSC sheets has been difficult without transmembrane proteins and scaffolds^32^ to maintain the structural integrity of the cell layer^33^.

This problem could be overcome by engineering with proteins, particularly cell-adhesive molecules such as fibronectin, collagen, or other ECM proteins, which are known to foster the adhesion and survival of stem cells^34,35^. In particular, fibronectin primarily mediates cell adhesion through heterodimeric integrin receptors, including α5β1, which binds to arg-gly-asp (RGD) and adjacent sequences in the central cell binding domain^36,37^. Cell adhesion to ECM proteins has been shown to affect the expression, localization, composition, and function of cell-cell interactions^38^. Tight coordination derived from these integrin-mediated cell adhesions is required during tissue morphogenesis to foster the formation of a functional, multicellular structure or a sheet-like organ^39,40^.

Herein, we report a photothermal method for harvesting of transferrable, free-standing hADSC sheets. The effect of soluble fibronectin on the cell proliferation rate was first examined. We then examined the effect of stem cell concentration on cell-cell interactions for sheet formation and harvesting as a transferable cell sheet. Finally, we showed the potential delivery of stem cell sheets in an animal model for full-thickness wound healing.

## Results and Discussion

### Preparation of the photothermal polymer surface for hADSCs

**Figure 1:**
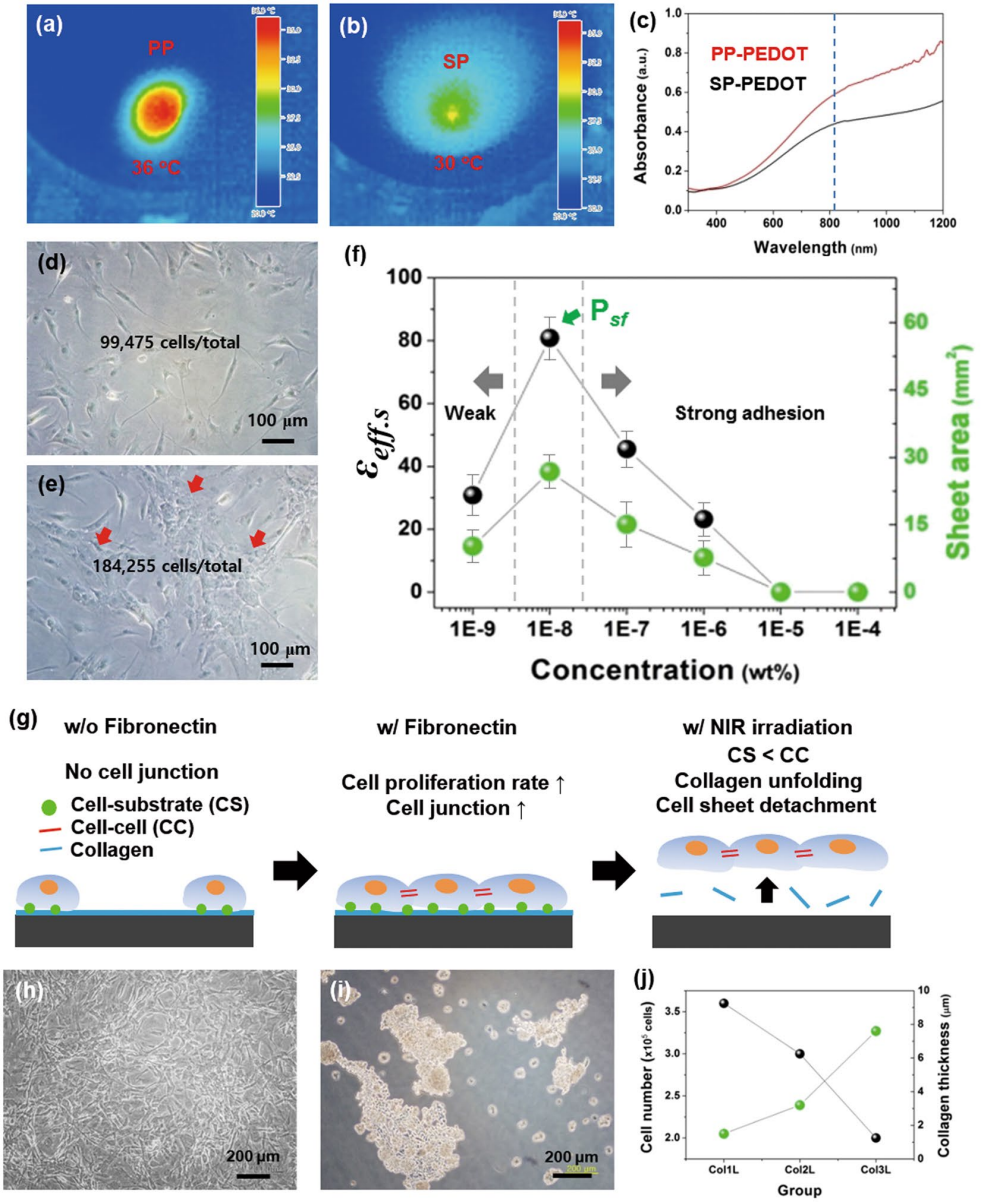
Culture conditions on CPP-PEDOT substrate with soluble fibronectin factor for hADSC sheet formation. Photothermal images of PP-PEDOT (**a**) and SP-PEDOT (**b**) in the same conditions (I_pw_ = 2.7 W/cm^2^, t_nir_ = 3 min, RD = 41.4J). (**c**) UV/Vis/NIR spectrum of PP-PEDOT (red) and SP-PEDOT (black) substrate (red arrow means 808 nm). After the cells were cultured for 1 day (cell seeding number = 100,000 cells/dish), optical images of the adherent hADSCs on the CPP-PEDOT substrate (**d**) without soluble fibronectin and (**e**) with soluble fibronectin (red arrows mean areas of concentrated cells). (**f**) Effect of harvesting efficiency (ε_d_) and sheet area (*A*_hcs_) at different fibronectin concentrations (1 μg/ml to 1 pg/ml, P_sf_ means the point of sheet formation). (**g**) Schematic image of the function of soluble fibronectin in hADSC culture conditions. (**h–j**) Culture conditions on CSP-PEDOT substrate for hADSC sheet formation. (**h**) Optical image of the adherent hADSCs on the CPP-PEDOT substrate at 1 day. (**i**) Optical image of the detached sheet fragments from the CPP-PEDOT substrate at 1 day. (**j**) Effect of the cell adhesion rate and the thickness of collagen layer.

A photothermal polymer film (PP-PEDOT) was prepared on a tissue culture polystyrene dish (TCPS) through a solution casting polymerization (SCP) process using a solution containing EDOT, pyridine, a polymeric surfactant (PEPG), and Fe(III) p-tosylate, similar to the polymer film (SP-PEDOT) for the formation of a human dermal fibroblast sheet^25^. The prepared PEDOT-coated substrate went through 5 times of washing step (dipped in fresh ethanol for 2 h and washed out) to remove any residual impurities. Upon drying the TCPS was coated with intrinsic PEDOT (PP-PEDOT). To demonstrate the photothermal properties, a NIR was exposed to the PEDOT face. Among the PEDOT samples, PP-PEDOT showed enhanced absorbance at 808 nm (Fig. 1c) and showed a higher temperature rise (36 °C) than that of SP-PEDOT (30 °C) at the same NIR exposure condition with an input power density (I_pw_) of 2.0 W/cm^2^, irradiation time (t_nir_) of 1 min, and radiation dose (RD) of 31.2J (Fig. 1a,b). The photothermally heated area (*A*_*PT*_) of PP-PEDOT was larger (*A*_*PT*_ = 0.2 cm^2^) than SP-PEDOT (*A*_*PT*_ = 0.01 cm^2^), possibly due to the increased photothermal efficiency of the former. Furthermore, the surface of PP-PEDOT was smoother than that of SP-PEDOT, as previously reported^41^. Thus PP-PEDOT (200 nm) was selected as a photothermal layer and coated on the TCPS dish. Then collagen layer (6.5 um) was coated on the PEDOT surface, which is abbreviated as CPP-PEDOT hereafter.

### Engineering of soluble FN for the formation of robust hADSC cell interactions and harvesting of hADSC sheets

We have previously shown that collagen-coated PEDOT substrates are effective for the preparation of a human dermal fibroblast sheet^25^. Contrary to the human dermal fibroblasts cells, which were adhered and were homogeneously distributed on the collagens coated PEDOT surfaces, the hADSCs were not evenly adhered onto the CPP-PEDOT surfaces and left a partially aggregated mass when the hADSCs were seeded at 4 × 10^5^ cells/dish. Thus, the cultured hADSCs on CPP-PEDOT for one day (Fig. 1h) afforded unstable and fragmented sheet pieces (Fig. 1i) by the photothermal detachment method. The hADSCs that were cultured for a longer period (3 days) were not detached from the CPP-PEDOT surface. This could be attributed to poor cell-to-cell (CC) and cell-to-substrate (CS) interactions, which are known to be extremely sensitive to the microenvironment of a so-called ‘niche’^30,31^. To improve the C-C and C-S interactions on the CPP-PEDOT surface and to create a transferrable cell sheet, hADSCs were seeded in cell culture media containing fibronectin molecules (FNs), which are known as cell adhesion molecules (CAMs). When the hADSCs was seeded for one day at a cell number of 100,000 cells/dish, cells were well proliferated and formed a confluent layer. The cell proliferation rate (*k*_*pr*_) in the presence of FNs was 184,255 cells/dish, which is higher than the rate without FNs (*k*_*pr*_ = 99,475 cells/dish) (Fig. 1d,e).

### Harvesting of hADSC sheets

To harvest the cell sheets, CPP-PEDOT substrates covered with confluent hADSCs were irradiated from the bottom face of TCPS with a coherent NIR laser (λ = 808 nm, *I*_pw_ = 2.7 W cm^−2^). The temperature at the CPP-PEDOT, increased up to 43 °C which is above the temperature of dissociation of collagen^25,26^ leading cell sheet detachment as shown in Fig. 1g. Since most of the light is absorbed by PEDOT (transmittance of 808 nm light <5%, Fig. 1c) and collagen layer is in-between cells and PEDOT film, cells are protected from direct exposure to a NIR source. As the collagens of triple helix structure in the film become unfolded into single strands at the light exposed area (43 °C), they are dissolved into solution media and the CS interactions are lost^25,26^. On the contrary, the CC interactions would stay intact to maintain as a sheet form during the harvesting of sheets due to CAM (Fig. 1g). As observed before, the temperature of the cell medium at the same condition was 37~38 °C that could not damage cells within short NIR irradiation time (<10 min). For this reason, the harvested cell sheets showed a high viability after harvesting by photothermal method^25,26^.

To find an optimized FN concentration for cell interactions and harvesting of a sheet, the area of the PEDOT and the cell concentration were fixed as 132.7 mm^2^ and 4 × 10^5^/dish, respectively. When the FN concentration was extremely high (10^−4^ to 10^−5^%; 1μg~100 ng/ml), cell sheets were not detached, even after 5 min of NIR exposure. This is probably due to the strong adhesion of the hADSCs on the CPP-PEDOT surface. With a dilute FN concentration (10^−6^ to 10^−9^%; 10 ng/ml ~10 pg/ml), the hADSC sheets were detached from the border of the CPP-PEDOT and floated onto the media after 3 min of NIR exposure. The sheet was completely detached with a harvesting efficiency (ε_d_) of 100% (Table 1) at a FN concentration from 10 ng/ml to 10 pg/ml:

1 $${\varepsilon }_{d}={A}_{det}/{A}_{nir}\times 100$$

where *A*_det_ and *A*_nir_ are the areas of the detached cells and NIR exposure, respectively. *A*_nir_ was the same as the area of CPP-PEDOT (132.7 mm^2^) because the temperature rise mainly occurred at the PEDOT film^26^.

**Table 1:** Culture conditions of the hADSC sheet on the CPP-PEDOT substrate with different concentration of soluble fibronectin factor and cells.

FN concentration^a^	Cell concentration	*A*_*det*_ (≤*A*_*nir*_)^b^ [mm^2^]	*ε*_*eff*.*d*_^c^ [%]	*A*_*hcs*_^d^ [mm^2^]	*ID*_*det*_^e^ [μm]
Control	4 × 10^5^	132.7	100	1.89	—
10^−4^% (1 μg/ml)	4 × 10^5^	0	0	0	—
10^−5^% (100 ng/ml)	4 × 10^5^	0	0	0	—
10^−6^% (10 ng/ml)	4 × 10^5^	132.7	100	7.74	—
10^−7^% (1 ng/ml)	4 × 10^5^	132.7	100	15.1	—
10^−8^% (100 pg/ml)	4 × 10^5^	132.7	100	26.8	13.7
10^−8^% (100 pg/ml)	8 × 10^5^	132.7	100	63	13.1
10^−8^% (100 pg/ml)	1.2 × 10^6^	132.7	100	72.8	12.6
10^−8^% (100 pg/ml)	1.4 × 10^6^	471.5	100	122.6	13.5
10^−9^% (10 pg/ml)	4 × 10^5^	132.7	100	10.2	—

^a^Concentration of fibronectin protein, ^b^detached area of PEDOT substrate, ^c^harvesting efficiency of detached area, ^d^detached area of hADSC sheet, ^e^intercellular distance of detached hADSC sheet. All sample were detached at the same input power density (2.7 W cm^−2^) and irradiated area of NIR laser (132.7 mm^2^).

**Figure 2:**
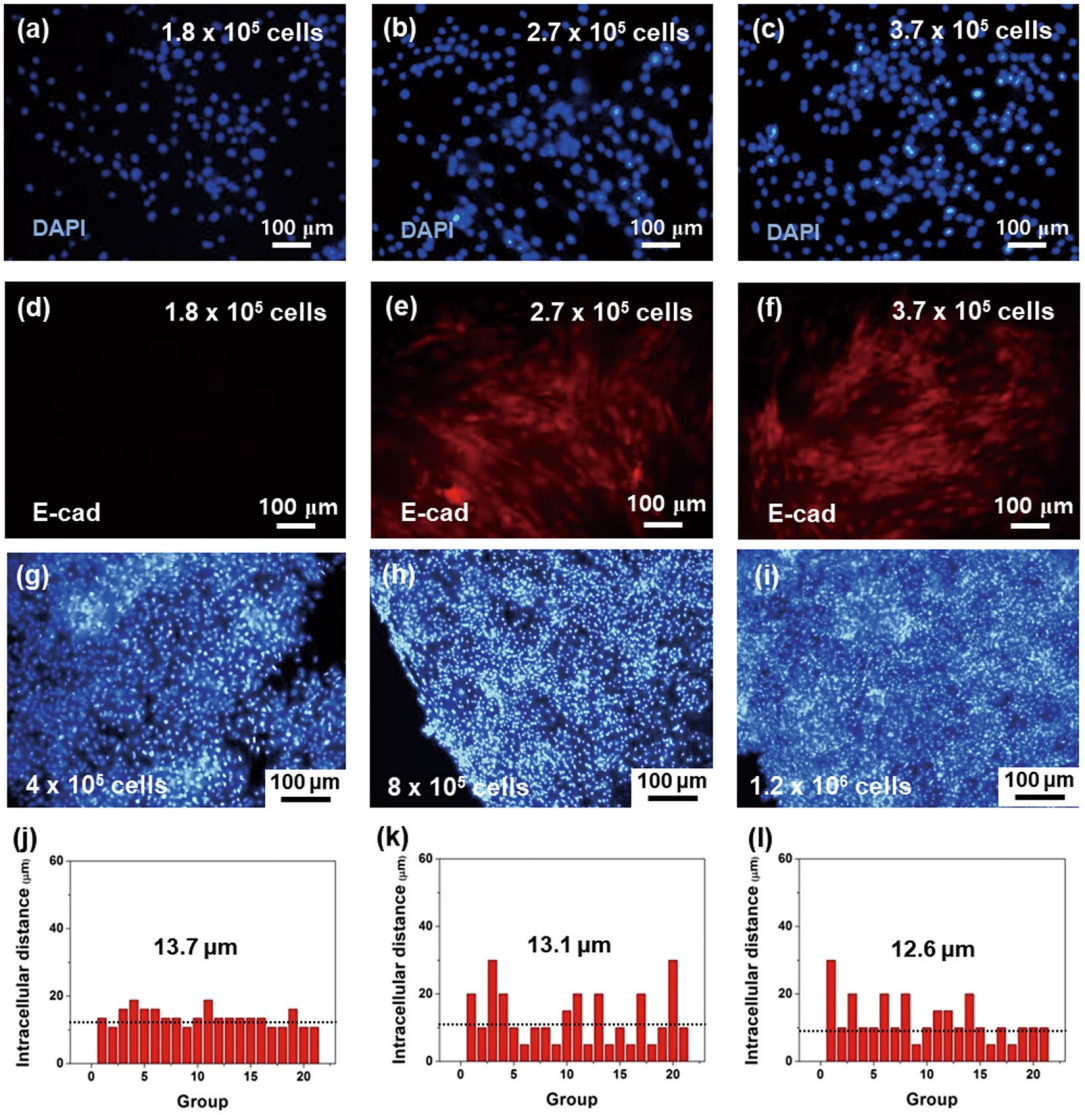
E-cadherin expression of the adherent hADSCs on the CPP-PEDOT substrate and intercellular distance of the harvested cell sheets at different cell concentrations. With 10^−8^% (100 pg/ml) soluble fibronectin molecules, DAPI/E-cadherin images of the adherent hADSCs on the substrate at different cell concentrations; cell seeding numbers were (**a**,**d**) 1.8 × 10^5^ cells/dish, (**b**,**e**) 2.7 × 10^5^ cells/dish, and (**c**,**f**) 3.7 × 10^5^ cells/dish. DAPI-stained images of the harvested sheets with different cell seeding numbers: 400,000 cells/dish (**g**), 800,000 cells/dish (**h**), and 1,200,000 cells/dish (**i**). Graphs of the calculated intercellular distance from the DAPI data at 400,000 cells/dish (**j**), 800,000 cells/dish (**k**), and 1,200,000 cells/dish (**l**). The cell groups are randomly selected cell groups in the same area of the cell sheet. Each cell groups in x-axis are consisting of two cells to measure intercellular distance between the two cells(y-axis).

Although the ε_d_ values were 100%, the harvested sheet area (*A*_hcs_) was always smaller than the *A*_det_ and *A*_nir_. This could be attributed to 1) the fact that the cells were not in a confluent state or had weak cellular interactions, resulting in fragmented sheets, and/or 2) the shrinkage of the cell sheet, which is often observed in cell sheet harvesting. Importantly, the harvested sheet area was strongly dependent on the FN concentration at the same cell concentration (4 × 10^5^ cells/dish), as shown in Fig. 1g and Table 1. When the FN concentration decreased from 1 μg/ml to 100 pg/ml, *A*_hcs_ increased and was maximized at the FN concentration of 100 pg/ml, after which *A*_hcs_ decreased significantly. The maximum *A*_hcs_ at 100 pg/ml reached 26.8 mm^2^ at a cell concentration of 4 × 10^5^ cells/dish. This result indicates that the soluble CAMs (FN) could enhance the cell proliferation rate and bring the cells in close proximity to one another and to the substrate (CPP-PEDOT) (Fig. 1g). It has been reported that the displacement of cell-cell (CC) interactions, enhancement of cell proliferation, and migration of cells could occur upon contact of the intercellular junctions with CAMs (e.g., FN, collagen, and laminin)^40^. From the DAPI (4′,6-diamidino-2-phenylindole) staining experiments, the cell proliferation number was counted at different cell concentrations under a fixed FN concentration (100 pg/ml). It was observed that the cells were concentrated and resided in proximity to one another as the cell number increased to 3.7 × 10^5^ cells/dish (Fig. 2a–c).

As shown in Fig. 1e, the CC interactions in the proliferated cells in the media containing FN went through re-formation of the CC interactions (Figs 1e and 2). E-cadherin expression was observed by fluorescence microscopy in the cell media with more than 2.7 × 10^5^ cells/dish (Fig. 2b,e). As the cells were further concentrated, the cell junctions increased and were vividly observed at a cell number of 3.7 × 10^5^ cells/dish (Fig. 2c,f). Thus, it was important to have a cell number larger than 3.7 × 10^5^ cells/dish to reach a concentrated state with a large number of intercellular junctions.

**Figure 3:**
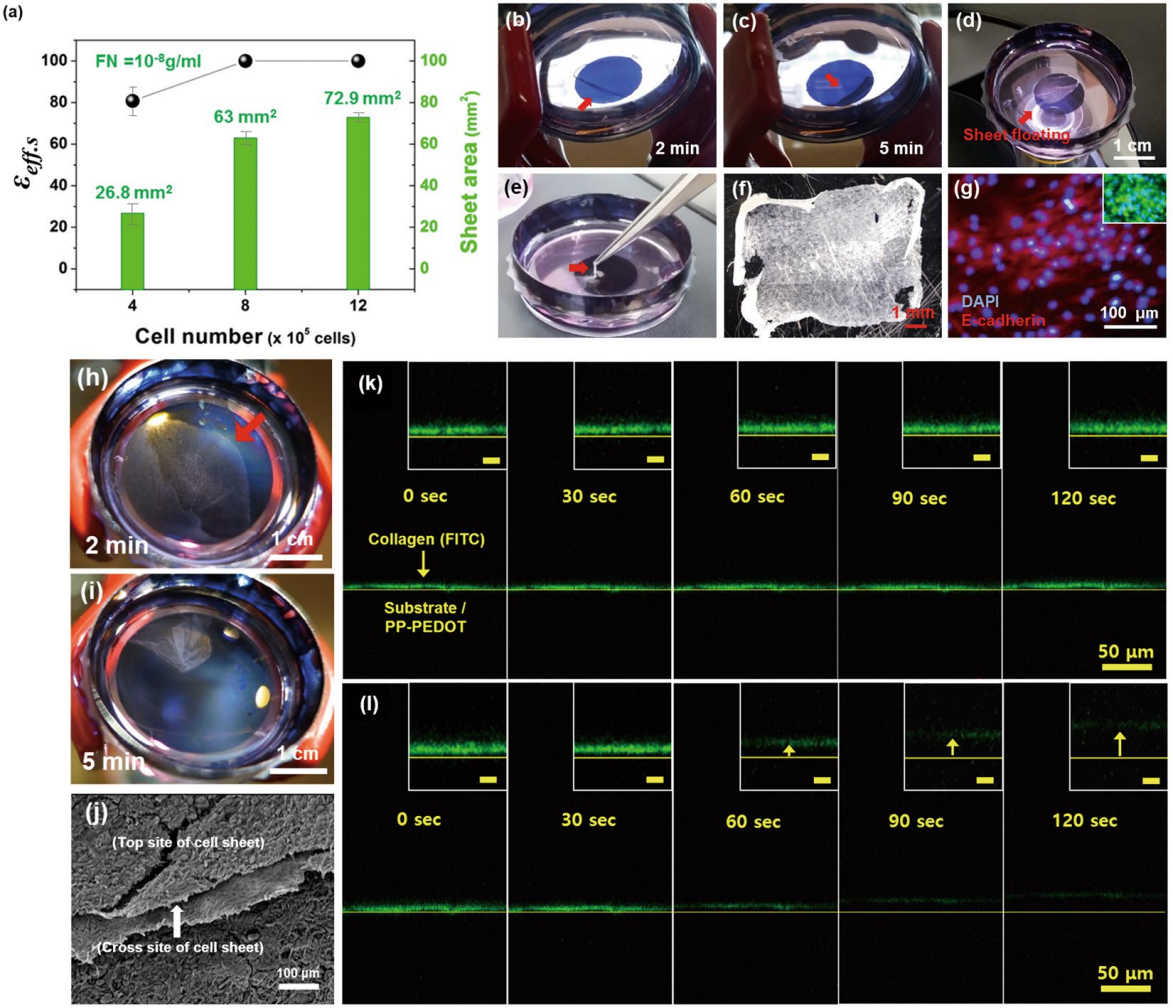
Sheet harvesting process of the hADSCs from the CPP-PEDOT substrate. (**a**) Effect of the harvesting efficiency (ε_d_) and sheet area (*A*_*hcs*_) at different cell concentrations. Optical microscope images of the detached sheets at 2 min (**b**), 5 min (**c**), and 10 min (**d**). (**e**) Digital camera image of the lifted sheet using the tiny tweezer. (**f**) Optical microscope image of the harvested sheet from the CPP-PEDOT substrate (1.3-cm circle pattern). (**g**) Fluorescent images of E-cadherin expression and live/dead assay (inset) of the harvested sheet. Optical microscope images of large cell sheet detachment with optimized conditions after NIR laser for (**h**) 2 min and (**i**) 5 min. (**j**) The cross-cut images by FE-SEM to identify the morphology of the cells in the harvested hADSCs sheet. The image from confocal microscopy with fluorescein isothiocyanate (FITC) as a fluorophore to trace the collagen (λ_exi_ = 495 nm, λ_emi_ = 519 nm) without (**k**) and with (**l**) NIR irradiation at different times. The light green region indicates a high concentration of collagen-FITC. (Scale bar: 50 μm) (Inset: magnified image, scale bar: 10 μm).

To investigate the optimum cell concentration for the formation of a full hADSC sheet, the hADSCs were cultured on the CPP-PEDOT substrate with different cell concentrations (4, 8, 12 × 10^5^ cells/dish) (Fig. 3 and Table S1). After the cells were cultured for 1 day, the cell sheets were obtained by the photothermal method under NIR exposure (I_pw_: 2.7 W/cm^2^, t_nir_: 10 min). Interestingly, the size (*A*_hcs_) of the harvested sheets increased as the seeded cell number increased (Fig. 3a) at the same *A*_nir_ (area = 132.7 mm^2^, d = 1.3 cm). Figure 2g–i show the DAPI staining results for the harvested cell sheets. The average nucleus distance of the neighboring cells (*d*_*nn*_) was identified by DAPI. The *d*_*nn*_ value slightly decreased in the harvested cell sheet as the cell number increased; however, it was not proportional to the seeded cell number. The intercellular distances in harvested cell sheet from the DAPI data, were calculated and exhibited with different cell seeding numbers (4 × 10^5^, 8 × 10^5^, 1.2 × 10^6^ cells/dish). The cell groups are randomly selected cell groups in the same area of the cell sheet. Each cell groups are consisting of two cells to measure intercellular distance between the two cells. The average intercellular distances of cells (groups) were displayed in Fig. 2j–l. As the distance at 4 × 10^5^ cells/dish was approximately 13.7 μm (Fig. 2j), an expected *d*_*nn*_ in the cell sheet harvested from 8 × 10^5^ cells/dish would be 6.9 μm assuming a proportional shrinkage of the cell sheet to cell number; however, the *d*_*nn*_ of the 8 × 10^5^ cells/dish concentration was determined as 13.1 μm by DAPI (Fig. 2k). The distance only slightly decreased with an increase in concentration, even under a high cell seeding condition such as 1.2 × 10^6^ cells/dish (*d*_*nn*_ = 12.6 μm). Instead of shrinkage, the harvested cell sheet area dramatically increased as the cell number increased. Thus, at an optimum FN concentration and cell seeding number, sheet shrinkage could be minimized, providing a large cell sheet.

Finally, at the seeded cell number of 12 × 10^5^ cells/dish, a full hADSC sheet was harvested by the photothermal dissociation of collagens on the CPP-PEDOT. Figure 3 shows the entire process of hADSC sheet harvesting. When the hADSCs were exposed to a NIR laser (λ: 808 nm, I_pw_: 2.7 W/cm^2^) for 2 min, the cells were partially detached and floated from the CPP-PEDOT substrate (Fig. 3b). After NIR exposure for 5 min, the cell sheet was almost detached and floated from the substrate (Fig. 3c,d). The harvested sheet was free-standing and was transferrable using tweezers (Fig. 3e). The sheet was highly dense, and the size of the harvested sheet was approximately 72.9 mm^2^ (Fig. 3f). E-cadherin expression (red) was observed in the cell sheet, and the cell viability was approximately 90%.

To obtain large-area cell sheets for *in vivo* chronic wound-healing experiments, the area of the CPP-PEDOT substrate was increased to 471.5 mm^2^ and hADSCs (1.4 × 10^6^ cells/dish) were seeded on the large CPP-PEDOT substrate with an optimized concentration of FN (100 pg/ml). After culturing the cells for 1 day, a large cell sheet was detached (Fig. 3h) and floated on the surface of the media (Fig. 3i) from the photothermal method using a NIR laser (*I*_*pw*_: 2l7 W/cm^2^, t_nir_: 2 min and 5 min, respectively). The *ε*_*eff*.*d*_ of the detached cell sheets in each condition was also 100%, and the detached area of the hADSC sheet was 122.6 mm^2^, which was a suitable area for chronic wound-healing applications (Table 1).

### Chemical analysis of the harvested cell sheet and media

Before the wound-healing application, the viability of the harvested cell sheet was further examined to identify any remaining toxic impurities, including (1) collagens from the CPP-PEDOT and (2) chemicals, such as iron and the monomers used for the preparation of PEDOT. To identify the remaining collagen in the harvested hADSC sheet, a sheet was harvested from the fluorescein isothiocyanate (FITC)-stained collagen layer that was coated on the PEDOT surface. Before NIR exposure, the FITC-stained collagen was detected with green fluorescence (Fig. 3k,l). Upon exposure to the NIR light source, the fluorescence intensities between the cell sheet and PP-PEDOT decreased within 2 min (Fig. 3l). This result is attributed to the photothermal dissolution of the collagen layer, in which collagens of insloluble triple helix structure were unfolded into soluble single strands upon phothermal heating, then dissolved out into ECM media. Before NIR irradiation, the collagen layer was not dissolved into the culture medium and the cell sheet was not floated from the CPP-PEDOT, as shown in Fig. 3k,l. After NIR irradiation, the collagen dissociation was started by the photothermally generated heat from the PP-PEDOT face. As NIR irradiation time goes by the distance between cell sheet to PP-PEDOT increased to 5.7 μm (60 sec), 9.8 μm (90 sec), and 14.7 μm (120 sec) (Figs 3k,l and S3). Finally, the fluorescence from FITC was almost undetectable in the harvested cell sheet (Figs 3k,l and Movie S1), indicating that the sheet is unlikely to transfer collagen from CPP-PEDOT.

To trace the iron ion (Fe^3+^) from the oxidant, dipped CPP-PEDOT was analyzed by an inductive coupled plasma mass spectrometer (ICP-MS). The PEDOT-coated substrate, which had gone through the washing step 4 times (dipped in fresh ethanol for 2 h and washed out), showed only a trace amount of Fe components. This metal content of Fe quantity (18 ng mL^−1^) from the 4-times repeated washing step was much lower than the content in the cell medium (284 ng mL^−1^) (Table S3). On the other hand, the Fe^3+^ quantity in the solution of the PEDOT that was dipped for 1 and 2 h followed by washing with ethanol was higher (1 h: 4837 ng mL^−1^, 2 h: 6328 ng mL^−1^) compared with that of the cell medium. This result confirmed that the PP-PEDOT substrate purified by the washing step may be suitable as a cell culture media and does not have the problem of residual Fe^3+^ ions.

Figure S4 shows Fourier transform infrared spectrometer (FT-IR) of the fully washed PP-PEDOT (blue line). The peak at 3115 cm^−1^ for EDOT monomer (Fig. S4, black) was due to the 2,5-hydrogen atoms on the thiophene ring^42^. The peak at 3115 cm^−1^ disappeared in the fully washed PP-PEDOT (blue line). Furthermore, the peaks from oxidants due to S-O stretching peaks (810 and 1006 cm^−1^) were not observed on the spectrum of PP-PEDOT after it was fully washed^43^. Therefore, for the fully washed PEDOT substrate, the FT-IR peaks of the monomer and oxidant were not observed, confirming that the ethanol washing process was highly suitable for PP-PEDOT without toxicity to cells.

Furthermore, to examine the stability of PP-PEDOT under NIR exposure during cell sheet harvesting, UV/Vis/NIR spectroscopy was used to determine any PEDOT residue in the harvested cell media. As shown in Fig. S5, there is no absorption at NIR range (700~900 nm) in the spectrum of the harvested cells in PBS obtained from the traditional trypsinization method (Fig. S5, PBS + cell (TCPS), blue line), because it was prepared without PP-PEDOT. Interestingly, the spectrum of the photothermally harvested cell sheet using CPP-PEDOT (PBS + cell sheet (PP-PEDOT), magenta) showed almost the same absorption spectrum as the sample obtained from the traditional trypsinization method. In addition, NIR absorption was not observed in the spectrum, even though it was prepared by PP-PEDOT. This result was strong evidence for using PP-PEDOT, as it was highly stable with NIR laser exposure for photothermal cell sheet harvesting. PP-PEDOT was not dissolved or decomposed in the medium during or after NIR exposure. Furthermore, PP-PEDOT did not remain in the harvested cell sheet. As a result, CPP-PEDOT and the photothermal cell sheet harvesting method are non-toxic, highly stable, and suitable for wound-healing applications.

### hADSC sheets for chronic wound healing

The potential use of the hADSC sheet for chronic wound healing was examined *in vivo* using genetically diabetic *db*/*db* mice. Firstly, we carried out the wound healing effect by the cells suspension injection group (cells alone group) and cell sheet treated group for normal mice prior to the db/db mice. The cell sheets treated group showed higher wound healing effect than the cells suspension injection group (Supplementary information, Fig. S6). This is in good agreement with the previous reports that cell sheets are quite effective for the chronic wound healing^44–46^.

**Figure 4:**
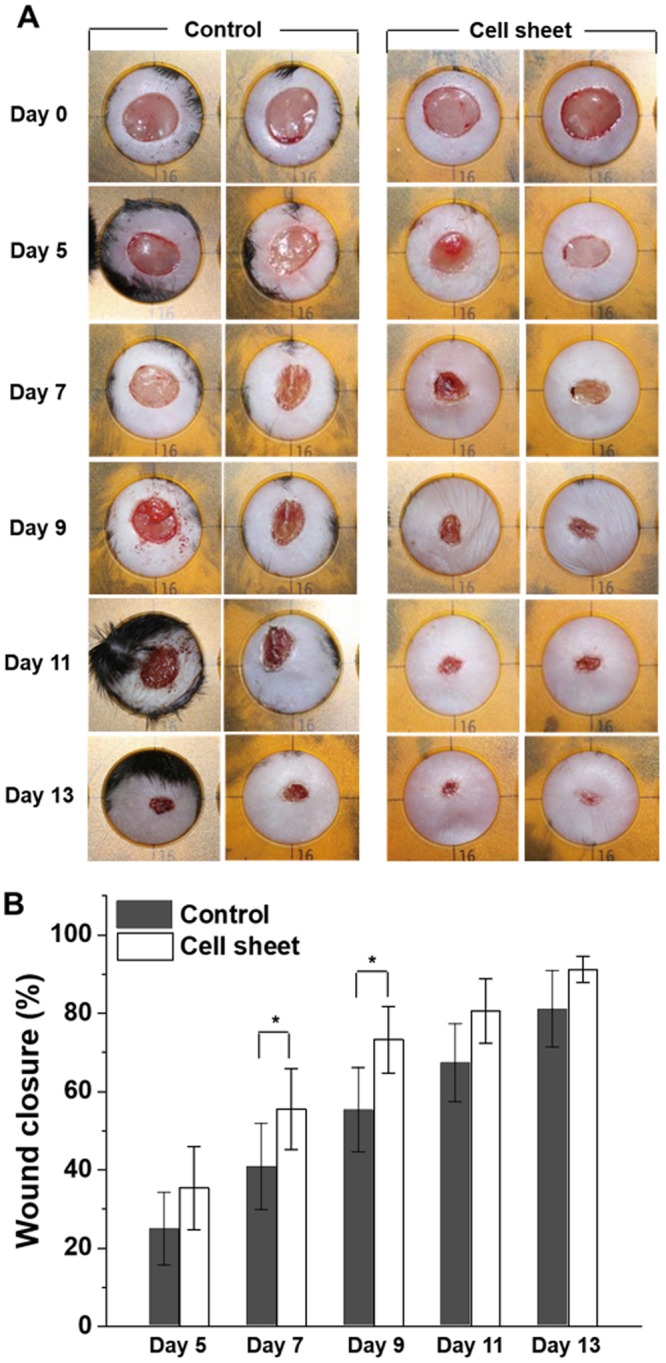
*In vivo* wound-healing process. A 50.2-mm^2^ size skin defect was created on the dorsum of diabetic *db*/*db* mice. (**a**) Representative photographs of wound healing with a 16-mm circular template after transplantations of the cell sheet at 0, 5, 7, 9, 11, and 13 days compared with the control. (**b**) Plot of the wound closure over time after hADSC sheet treatment (black) compared with the control (open). The LMM (linear mixed model) method was used to determine the wound size for confound symmetry covariance within mouse P-value with bonferroni correction. Each value represents the mean ± SD (n = 10). *P < 0.05 compared with the control.

A photothermally harvested hADSC sheet (50.2 mm^2^) containing 1 × 10^6^ cells was transferred onto the wound (area = 50.2 mm^2^). The free-standing hADSC sheet was transferred and attached onto the wound by virtue of the wet surface nature of the wound. In the control group (untreated), each wound was wrapped with PBS wet gauze for a while, and then the gauze was removed to reach a hydrated environment that was similar to the hADSC sheet-treated group. Figure 4 shows the progression of the wound-healing process over time. Treatment with a hADSC sheet on the skin wound resulted in more effective wound closure compared with the control (Fig. 4a). On the 5^th^ postoperative day, wound closure in the hADSC sheet-treated groups was similar to that of the control. However, at day 7, the hADSC sheet-treated group led to significantly faster wound closure with a wound closure effect (ε_wc_) of 55% compared with that of the control (ε_wc_ = 41%). The wound closure effect (ε_wc_) was determined as follows:

2 $${{\rm{\varepsilon }}}_{wc}\,( \% )={A}_{wc}/{A}_{iwa}\times 100$$

where *A*_wc_ and *A*_iwa_ are the wound closure area after treatment and the initial wound area (50.2 mm^2^), respectively.

Furthermore, at day 9, wound closure was much greater in the hADSC sheet-treated group (ε_wc_ = 73%) than in the control (ε_wc_ = 55%) (Fig. 4b). These results indicated the superior performance of the harvested stem cell sheets over the control in healing the chronic wound. As the stem cell sheet could be harvested from the non-invasive photothermal method within a very short time (5~10 min), the wound-healing properties of hADSC sheet could be designed to help pave the way for various stem cell therapies and organ regeneration.

## Conclusion

Transferrable and free-standing hADSC sheets were obtained via the photothermal method within few minutes by controlling the cell-cell (CC) and cell-substrate (CS) interactions. A soluble protein solution based on fibronectin (FN) was employed to enhance the CC interactions and the cell proliferation rate on the CPP-PEDOT. In the presence of FN molecules, cells were in contact with neighboring cells to promote CC and CS interactions, leading to full sheet formation. The cell proliferation rate and the harvested cell sheet area could be optimized by the combination of FN concentration (10^−8^%, 100 pg/mL) and cell seeding number (1.2 × 10^6^ cells) to produce a large area, transferrable, free-standing hADSC sheet (122.6 mm^2^) by the photothermal method. A hADSC sheet was transferred to a wound on diabetic *db*/*db* mice to observe the superior performance of the harvested stem cell sheets over the control. The wound closure with hADSC was faster than that of the control (30%↑). This result indicates that the hADSC sheet harvesting system can be applied to various therapies, such as skin reproduction and organ regeneration, with immense promise as an emerging strategy in tissue engineering.

## Materials and Methods

### Materials and Instruments

3,4-Ethylenedioxythiophene, iron-(III) p-toluenesulfonate, anhydrous isopropyl alcohol, fibronectin, and gelatin were purchased from Sigma-Aldrich Chemicals and were used without further purification. Acid-soluble collagen solution (3.0 mg/ml, 0.3 wt%), DMEM, fetal bovine serum (FBS), antibiotics (penicillin/streptomycin), phosphate buffered saline (PBS, pH 7.4), trypsin/EDTA (0.05%), and Trypan blue (0.4%) were purchased from Gibco (Invitrogen, Carlsbad, CA). Other chemicals and solvents were purchased from Sigma-Aldrich (St. Louis, MO). A high-power fiber-coupled NIR diode laser (808 ± 3 nm, 6.0 W, Real Light) was modulated by changing the applied current using a power supply, and a thermoelectric cooling module was used to cool the hot diode laser at high power modulated by the power supply. The focusing of the laser exposed area and power density of the laser were controlled by the distance between the end of the fiber and the PS substrate and collimator. The laser exposed area was determined by NIR fluorescent detection card (Edmond Optics). The thermal image and temperature distribution data were taken by a thermal image camera (875i, Testo). A confocal microscope (Axio imager Z2, LSM 700, Carl Zeiss) was used to obtain images of the side view. Fluorescein isothiocyanate (FITC)-stained collagen was used to trace collagen dissociation. A laser was used as the excitation source for FITC (λ_exi_ = 495 nm, λ_emi_ = 519 nm).

### Preparation of the CPP-PEDOT substrate for cell culture

The PP-PEDOT substrate was prepared with pyridine (13.54 mg) and PEPG triblock co-polymer (200 mg). They were added into 1 g of the oxidative solution containing 40 wt% of iron(III) *tris*-*p*-toluenesulfonate (Fe-Tos) in n-butanol. After stirring for 6 h, the solution was sonicated for 10 min to make a homogeneous solution. The coating solution consisted of 52.8 wt% of the mixture solution of pyridine, PEPG, Fe-Tos, monomer, and 47.2 wt% of n-butanol. The molar ratio of pyridine: iron(III) tosylate: monomer was fixed at 0.55: 2.25: 1. Then, it was cooled before adding the EDOT monomers. The oxidative solution containing EDOT was spin-coated onto the polystyrene petri dish substrates at 2000 rpm for 30 sec. All the samples were polymerized at 90 °C for 1 h. After cooling to room temperature, the conductive polymer-coated substrate was washed several times with 70 wt% ethanol to remove residual oxidant, low molecular weight oligomers, and impurities. The film was then dried under nitrogen flow. The patterned PP-PEDOT surface was obtained by the polymerization of a patterned monomer layer (including oxidant and pyridine), which was prepared by the removal of an unwanted area of the pattern using a cotton pencil that was wet with solvent. The patterned monomer layer (including oxidant and pyridine) was then subjected to polymerization at 60 °C. The prepared patterned PP-PEDOT substrate was washed several times with 70 wt% ethanol and was dried before collagen adsorption for the cell-detachment experiments.

### Cell collection

Under general anesthesia, tumescent was infiltrate into patients’ abdominal area. After 20 minutes, suction of abdominal fat was achieved through cannulas. This study was approved by the Institutional Review Boards of Severance Hospital of Yonsei University Health System. Briefly, adipose tissue was digested in type I collagenase solution (0.1%, Invitrogen, Carlsbad, CA, USA) with shaking in a 37 °C for 1 h. To stop the enzyme activity, growth medium was added to the digested tissue. And then, the tissue was filtered to remove large debris through a 70 μm cell strainer. Subsequently, the cell pellet was incubated with erythrocytes lysis buffer (Cambrex, Lonza, MD, USA) for 5 min at room temperature. After centrifugation, the stromal vascular fraction (SVF) was plated in DMEM containing 10% fetal bovine serum, 1% penicillin/streptomycin, and 5 ng/mL basic fibroblast growth factor (all from Invitrogen). Next day, non-adherent cells were removed and fresh complete medium was replaced. Adipose tissue-derived stem cells were cultured and expanded in a humidified atmosphere, 5% CO_2_ at 37 °C for study.

### Cell culturing and harvesting of stem cell sheets on CPP-PEDOT substrates

Human adipose-derived stem cells (hADSCs) were collected from healthy donors with approval from the Research Ethics Committee of Severance Hospital in Yonsei University, Seoul, Korea, (Approval No. 4_2008_0643) and informed consent. The cells were plated in DMEM supplemented with 10% FBS, 100 U/mL penicillin, and 100 μg/mL streptomycin at a density of 10^5^ cells/cm^2^ in a 75-cm^2^ tissue culture flask (Nunc, Denmark) at 37 °C in 5% humidified CO_2_. Non-adherent cells were removed after 24 h by exchanging the culture medium with repeated washing with PBS. The medium was changed every 3 days. Upon reaching 90% confluence, the hADSCs were recovered using 0.05% Trypsin/EDTA and were re-plated onto a CPP-PEDOT substrate. The CPP-PEDOT substrate was washed with 1× PBS buffer and was placed in a 35-mm culture dish (Corning Inc., NY). Next, the CPP-PEDOT substrate was coated with an aqueous solution of collagen (0.3 wt%) by drop-casting, washed out with distilled water, and sterilized with a UV treatment for 5 min. Cell counts were determined by counting the number of cells with a hemacytometer after Trypan blue staining in a counting chamber. To control the shape of the cell sheet, a circle-patterned PP-PEDOT substrate (diameter = 13 mm) was used.

After culturing for 1 day, CPP-PEDOT substrates with confluent cells were irradiated for 5 min with an NIR coherent diode laser (808 nm, 2.7 W cm^−2^). The shape and size of the detached cell sheets were observed using an Olympus inverted research microscope (Model IX71). Harvesting efficiency (ε_d_) of the detached sheet was defined as the ratio of the detached sheet size (*A*_*hcs*_) divided by the sheet-detached area (*A*_*det*_).

3 $${{\rm{\varepsilon }}}_{d}={\boldsymbol{sh}}\times \frac{{A}_{hcs}}{{A}_{det}}\times 100$$

For the shrinkage constant (*sh*) of the cell sheets, the cell-cell distance was calculated with DAPI staining before and after cell detachment. The distance was measured between center of cells with staining DAPI. Cells were randomly selected within the area of 200 × 200 μm^2^. It was measured 20 times at the nearest cell and the average value was calculated. The cells were analyzed via a live and dead assay to determine cell viability. The viability of the detached cell sheet by NIR exposure was analyzed using the Live/Dead viability kit (Molecular probes, cat no. L3224) according to the instructions of the manufacturer. Briefly, harvested cells were stained with phosphate-buffered saline (PBS) containing 2 μM of a calcein AM solution and 4 μM of an ethidium homodimer-1 solution for 30 min at room temperature in the dark. After washing with PBS, images were acquired using an Olympus inverted research microscope (Model IX71).

### Effect of sheet formation with soluble fibronectin molecules

For sheet formation, soluble fibronectin molecules were used as a cell adhesion molecule. Cells were seeded onto the CPP-PEDOT substrates at 4 × 10^5^ cells/dish with different soluble factor concentrations (fibronectin: 1 μg/ml to 1 pg/ml). The size of the detached cell sheets was calculated by a scale bar in the optical microscope, and the sheets were compared with those at other concentrations of the soluble fibronectin molecule. To study the effect of cell junction generation with soluble fibronectin, E-cadherin expression (Santa Cruz Biotechnology, cat no. sc-7870), which represents the protein of the cell junction, was observed by fluorescence microscopy at different cell concentrations.

### Characterization

An inductive coupled plasma mass spectrometer (ICP-MS, PerkinElmer: MexION300) was used to detect the dissolved Fe ions in the wash solution after washing the conductive polymer by analyzing the change of metal components according to time (1, 2 h) and number (4 times) to confirm the removal of residual metal ions. A Fourier transform infrared spectrometer (FT-IR, Bruker: Vertex70) was used to detect the unreacted monomer and oxidant after washing the PEDOT. From the measurement, the detected residual unreacted impurities were used to confirm the toxicity of PP-PEDOT. UV/Vis/NIR spectroscopy was measured to confirm the conductive polymer that was dissolved in the cell medium. After cell and cell sheet detachment, the solution samples were prepared with cell, cell sheets and medium in a quartz cell with an air reference.

### Anesthesia and creation of excisional wound

All experimental procedures performed in this study followed ethical guidelines for animal studies and were approved by the Institutional Animal Care and Use Committee of Yonsei University College of Medicine (IACUC No. 2016–0095). All experiments were performed in accordance with relevant guidelines and regulations.

Seven-week-old male genetically diabetic *db*/*db* mice were selected for a chronic wound-healing model. Twelve mice were randomly divided into control and experimental groups. We applied hair removal cream (Veet^®^, Cat. No. 20070747) on the dorsal skin of anesthetized mice and completely removed the cream and hair after five minutes. A 50.2 mm^2^ size skin wound was made on the dorsal side using an 8 mm-biopsy punch. The skin flap was removed from the wound bed.

### Stem cell transplantation

For the hADSC sheet-treated group (n = 10), a 50.2 mm^2^ hADSC sheet containing 1.2 × 10^6^ cells was transferred on the wound by using a polyester container that was poured into the open weave of the polyester (Urgotul®, Laboratoires URGO, Dijon, France) with the medium. After the medium has drained through the mesh, the sheet was carefully transferred to the wound by removing only the polyester mesh after 1 minute. In the control group (n = 10), Urgotul® that was buried in PBS was put on the wound and removed as the sheet transfer process to make a hydrated environment similar to that of the hADSC sheet-treated group. Subsequently, all wounds were covered with Tegaderm^3M^ sterile transparent dressing.

### Wound analysis

We uncovered the wound dressings and took photographs of individual wounds every third day from the 5th day. A 16 mm-diameter circular template was used as a reference to analyze the size of the remaining wound. Using the ImageJ program, the size of the template and the remaining wound size were measured. We used Statistical analysis system (SAS) (version 9.2, SAS Inc., Cary, NC, USA) along with the LMM (linear mixed model) method for measuring and calculating the wound size for confound symmetry covariance within mouse P-value with bonferroni correction. Setting the first wound size as 100%, we calculated the time for the wound to reduce to 70%, 50%, 30%, and 10%.

## Electronic supplementary material

Supporting figures and tables

Collagen dissociation movie
