# Supplementary material for: Protein-Engineered Large Area Adipose-derived Stem Cell Sheets for Wound Healing
Source: Sci Rep. 2018 Oct 26;8:15869. doi: 10.1038/s41598-018-34119-x (PMC6203842; doi:10.1038/s41598-018-34119-x)
Supplement: Supplementary file 1 — Supporting figures and tables [file 41598_2018_34119_MOESM1_ESM.docx]

Supplementary information

**Protein-engineered large area adipose-derived stem cell sheets for wound healing**

*Jongbeom Na^1^, Seung Yong Song^2^, Jae Dong Kim^1^, Minsu Han^1^, June Seok Heo^3^, Chae Eun Yang^2^, Hyun Ok Kim^3^, Dae Hyun Lew^2,*^, and Eunkyoung Kim^1,*^*

*^1^* Department of Chemical and Biomolecular Engineering, Yonsei University, 50 Yonsei-ro, Seodaemun-gu, Seoul 03722, South Korea

E-mail: [eunkim@yonsei.ac.kr](mailto:eunkim@yonsei.ac.kr)

*^2^* Prof. S. Y. Song, C. E. Yang, Prof. D. H. Lew

Institute for Human Tissue Restoration, Department of Plastic & Reconstructive Surgery, Yonsei University College of Medicine, Seoul, South Korea

E-mail: [DHLEW@yuhs.ac](mailto:DHLEW@yuhs.ac)

*^3^* J. S. Heo, Prof. H. O. Kim
Cell Therapy Center, Severance Hospital, Yonsei University College of Medicine, Department of Laboratory Medicine, Yonsei University, 50 Yonsei-ro, Seodaemun-gu, Seoul 03722, South Korea


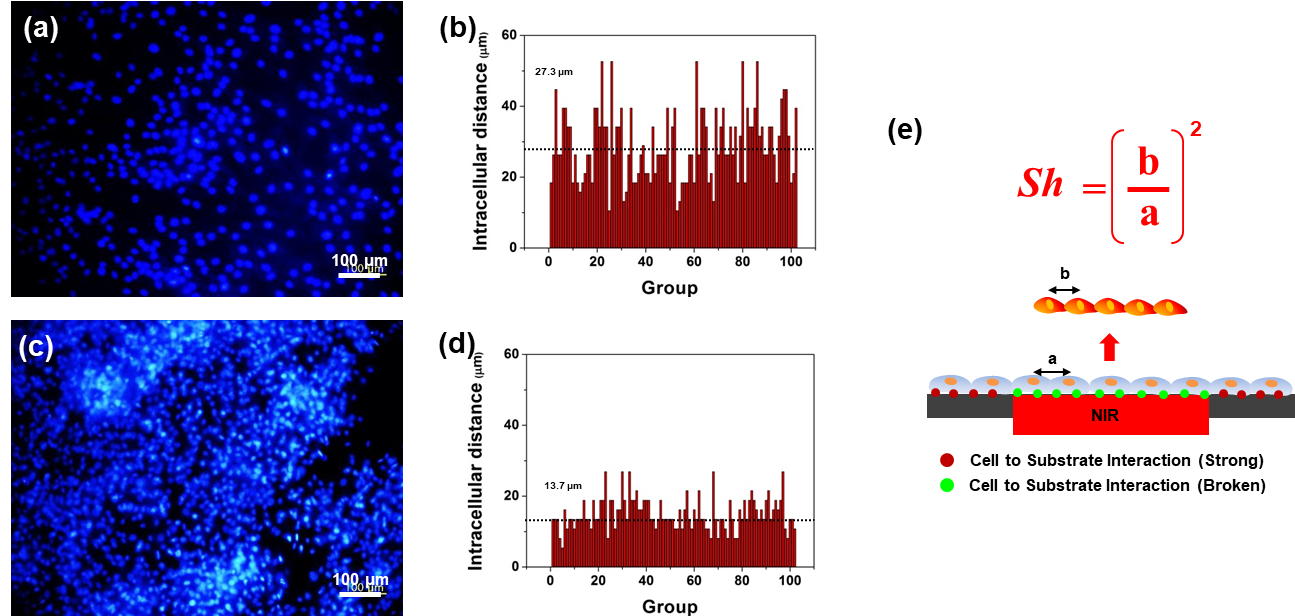


**Figure S1** Cell sheet shrinkage constant of the detached cell sheet from the **CPP-PEDOT** substrate. (a) DAPI staining image and (b) intracellular distance of the attached cells on the **CPP-PEDOT** (cell seeding number = 400,000 cells/dish). (c) DAPI staining image and (d) intracellular distance of the detached cells from the **CPP-PEDOT** (cell seeding number = 400,000 cells/dish). The cell groups are randomly selected cell groups in the same area of the cell sheet. Each cell groups in x-axis are consisting of two cells to measure intercellular distance between the two cells(y-axis). (e) Schematic image of cell sheet shrinkage constant of the detached cells (Sh = 0.25 at 400,000 cells/dish).


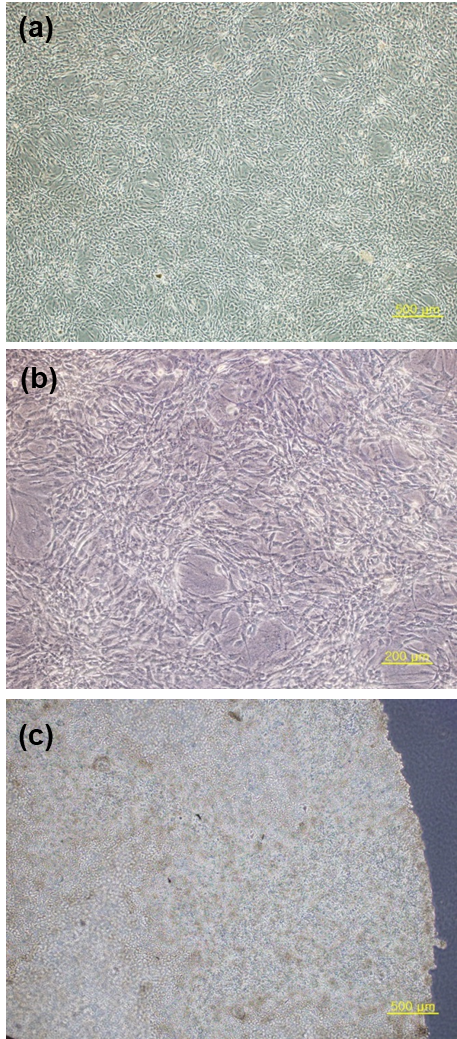


**Figure S2.** The optical microscopic images to observe the morphology of the hADSCs before and after NIR irradiation. (A) Optical microscopic image of **CPP-PEDOT** after cell culture (before NIR irradiation). (B, C) Optical microscopic image of **hADSC** sheet after detachment and transfer to a fresh petri-dish (C magnified to B).


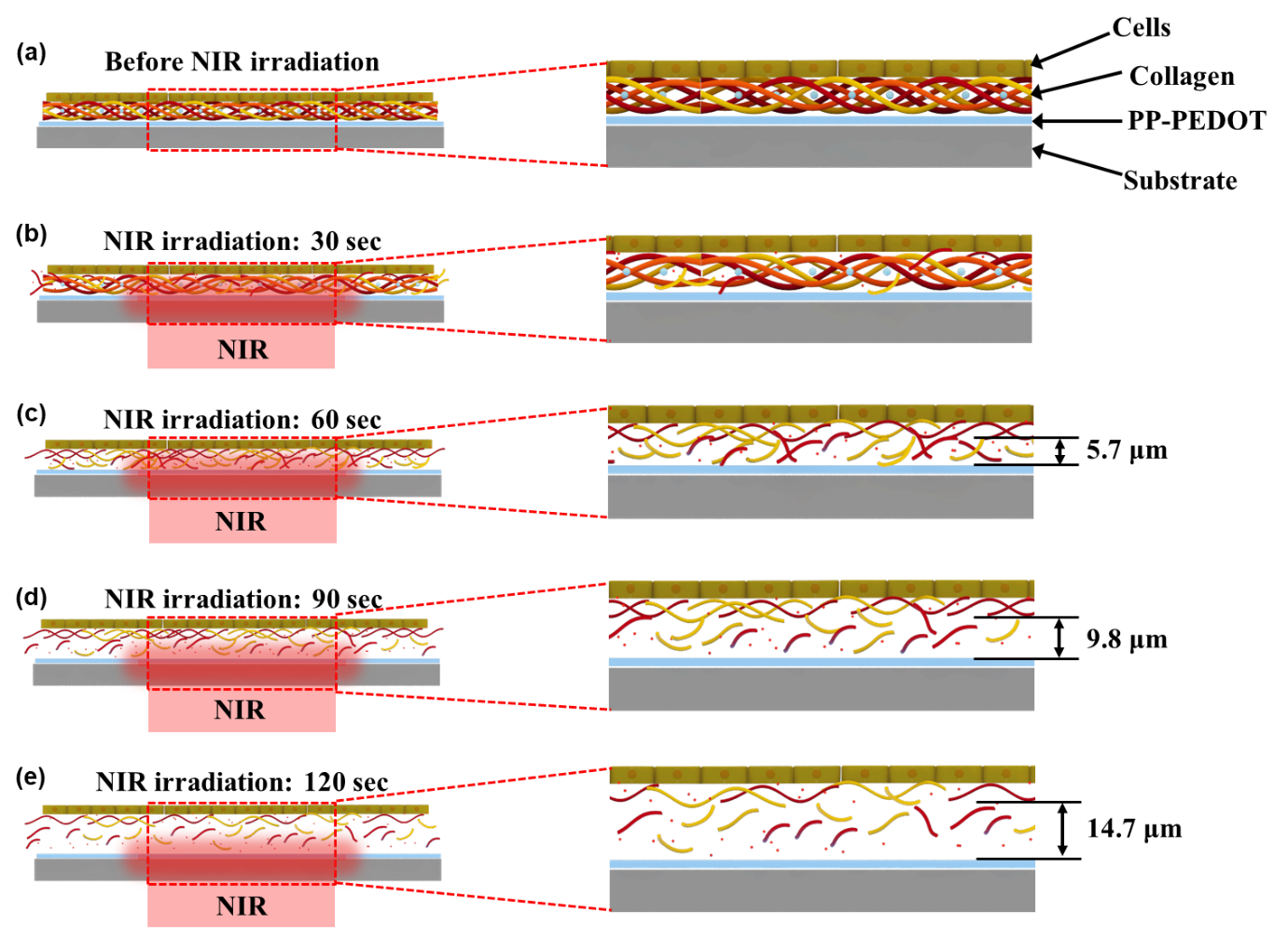


**Figure S3** A schematic illustration of cell sheet harvesting from the PP-PEDOT by photothermal method that depends on NIR irradiation time. (a) Before NIR irradiation, the cell sheet was not floated. (b) After NIR irradiation, the collagen dissociation was started by photothermal heat. As NIR irradiation time goes by the distance between cell sheet to PP-PEDOT increased to (c) 5.7 μm (60 sec), (d) 9.8 μm (90 sec), and (e) 14.7 μm (120 sec).


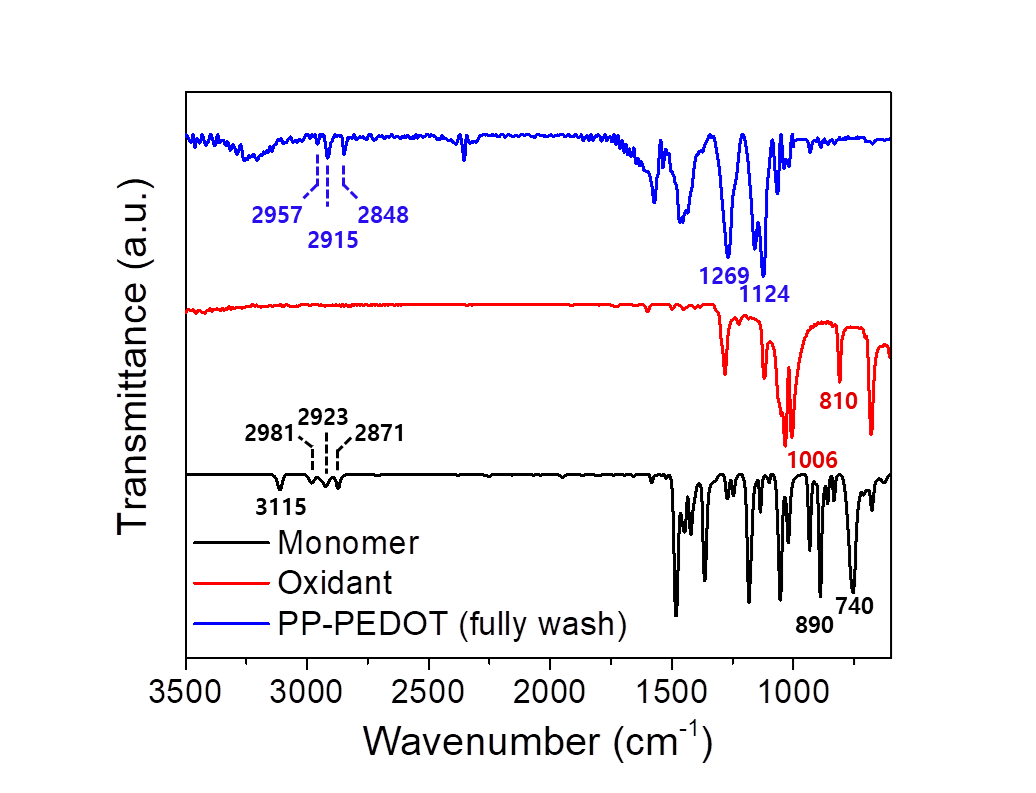


**Figure S4** The FT-IR spectrum of monomer (black line), oxidant (red line), and PP-PEDOT after fulling washing (blue line) to detect of residue materials. Monomer: 2,5-hydrogen atoms on the thiophene ring (3115 cm^-1^) Oxidant: S-O stretching (810 and 1006 cm^-1^)

**Figure S5** The UV/Vis/NIR spectrum of PBS (black), PBS+PP-PEDOT (red), PBS+cell (blue), and PBS+cell sheet (magenta) after NIR exposure, monitoring the PP-PEDOT absorption at NIR region: 700 ~ 900 nm.


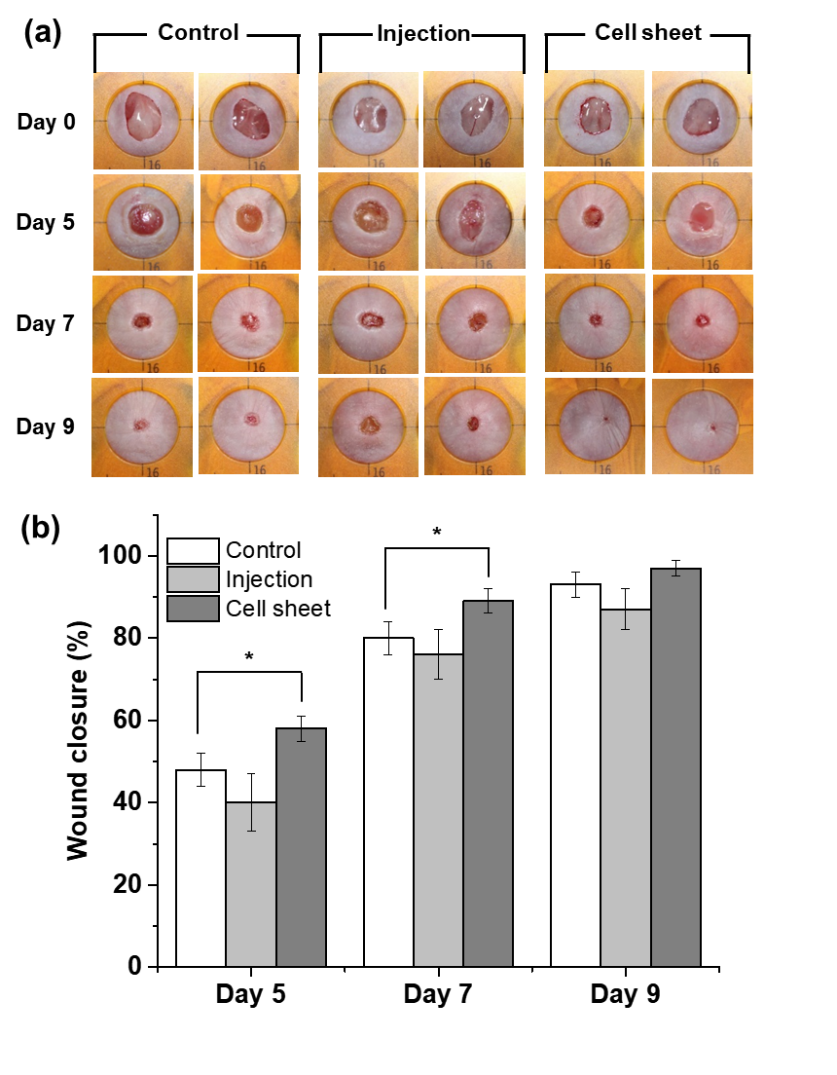


**Figure S6.** *In vivo* wound-healing process. A 50.2-mm^2^ size skin defect was created on the dorsum of SKH-1 hairless mice. (a) Representative photographs of wound healing with a 16-mm circular template after injection suspension cells and transplantations of the cell sheet at 0, 5, 7, and 9 days compared with the control. (b) Plot of the wound closure over time after injection of hADSC suspension (gray) and sheet treatment (dark gray) compared with the control (open). Each value represents the mean±SD. (n = 10) * P < 0.05 compared with the control.

**Table S1** Culture conditions of the **hADSC** sheet on the **CPP-PEDOT** substrate with different concentration of soluble fibronectin factor.

| No.^a)^ | *I_pw_* ^b)^  *­*[W cm^-2^] | *A_nir_* ^c)^  [mm^2^] | *A_det_ (≤ A_nir_)* ^d)^  [mm^2^] | *ε_eff.d_* ^e)^  [%] | *A_hcs_* ^f)^  [mm^2^] | *ε_eff.s_* ^g)^  [%] |
| --- | --- | --- | --- | --- | --- | --- |
| Control | 2.7 | 132.7 | 132.7 | 100 | 1.89 | 5.7 |
| 10^-4^ % (1 μg/ml) | 2.7 | 132.7 | 0 | 0 | 0 | 0 |
| 10^-5^ % (100 ng/ml) | 2.7 | 132.7 | 0 | 0 | 0 | 0 |
| 10^-6^ % (10 ng/ml) | 2.7 | 132.7 | 132.7 | 100 | 7.74 | 23.2 |
| 10^-7^ % (1 ng/ml) | 2.7 | 132.7 | 132.7 | 100 | 15.1 | 45.5 |
| 10^-8^ % (100 pg/ml) | 2.7 | 132.7 | 132.7 | 100 | 26.8 | 80.8 |
| 10^-9^ % (10 pg/ml) | 2.7 | 132.7 | 132.7 | 100 | 10.2 | 30.7 |

^a)^ Concentration of fibronectin protein, ^b)^ Input power density (W cm^-2^), ^c)^ irradiated area of NIR laser, ^d)^ detached area of **PEDOT** substrate, ^e)^ harvesting efficiency of detached area, ^f)^ detached area of human cell sheet, ^g)^ harvesting efficiency of human cell sheet. **PEDOT** area = 132.7 mm^2^

**Table S2** Culture conditions of the **hADSC** sheet on the **CPP-PEDOT** substrate with soluble fibronectin factor at different cell concentration.

| No.^a)^ | Cell concentration | *I_pw_* ^b)^  *­*[W cm^-2^] | *A_nir_* ^c)^  [mm^2^] | *A_det_ (≤ A_nir_)* ^d)^  [mm^2^] | *ε_eff.d_* ^e)^  [%] | *A_hcs_* ^f)^  [mm^2^] | *ε_eff.s_* ^g)^  [%] |
| --- | --- | --- | --- | --- | --- | --- | --- |
| Control | 4×10^5^ | 2.7 | 132.7 | 132.7 | 100 | 1.89 | 5.7 |
| 10^-4^ % (1 μg/ml) | 4×10^5^ | 2.7 | 132.7 | 132.7 | 100 | 26.8 | 80.8 |
| 10^-5^ % (100 ng/ml) | 8×10^5^ | 2.7 | 132.7 | 132.7 | 100 | 63 | 100 |
| 10^-6^ % (10 ng/ml) | 1.2×10^6^ | 2.7 | 132.7 | 132.7 | 100 | 72.9 | 100 |

^a)^ Concentration of fibronectin protein, ^b)^ Input power density (W cm^-2^), ^c)^ irradiated area of NIR laser, ^d)^ detached area of **PEDOT** substrate, ^e)^ harvesting efficiency of detached area, ^f)^ detached area of human cell sheet, ^g)^ harvesting efficiency of human cell sheet. **PEDOT** area = 132.7 mm^2^

**Table S3** Residual metal (Fe) ion concentration after washing process of **PP-PEDOT**.

| Sample ^a)^ | Metal (Fe) ion concentration [ng mL^-1^] |
| --- | --- |
| 1 time (1 h) ethanol washing | 4837 |
| 2 times (2 h) ethanol washing | 6328 |
| 4 times (4 h) ethanol washing | 18 |
| Cell medium | 284 |

^a)^ All samples were prepared from the solvent that was used washing process at last time.
